# Rapid emergence of *FKS* mutations in *Candida glabrata* isolates in a peritoneal candidiasis

**DOI:** 10.1016/j.mmcr.2017.04.004

**Published:** 2017-04-25

**Authors:** Milène Sasso, Claire Roger, Laurence Lachaud

**Affiliations:** a Laboratoire de Parasitologie-Mycologie, Centre Hospitalo-universitaire de Nîmes, Nîmes, France; b Service de réanimation chirurgicale, Centre Hospitalo-universitaire de Nîmes, Nîmes, France; c Département de Parasitologie-Mycologie, Centre Hospitalo-universitaire de Montpellier, France

**Keywords:** Echinocandin resistance, *Candida glabrata*, Antifungal susceptibility testing, *FKS* mutations, MIC interpretation

## Abstract

We report a rapid acquisition of echinocandin resistance after 12 days of micafungin treatment, without prior exposure, in a patient with peritoneal candidiasis due to *C. glabrata*. Isolates recovered before and after treatment were compared by multilocus sequence typing. Results of antifungal susceptibility testing and *FKS* mutations were reported. The interest of repeating antifungal susceptibility testing for echinocandin molecules during the treatment is discussed and a strategy to research *FKS* mutations proposed.

## 1 Introduction

International recommendations for the treatment of invasive candidiasis are now based on an echinocandin molecule as the first line of antifungal therapy [1], [2]. This recommendation is well adapted to the second most isolated species, *Candida glabrata*, because of its lesser susceptibility to azole drugs. Intrinsic resistance to echinocandins is rare and mainly observed for *Candida parapsilosis* [3]. Nevertheless, the wide use of echinocandins has led to a decrease in susceptibility or to resistance acquisition in *Candida* strains, mainly for *C. glabrata*. The resistance is conferred by the presence of mutations in hot-spot regions of the *FKS1* and *FKS2* genes encoding β-1,3-D-glucan synthase, the echinocandin target enzyme [4]. Resistances appear after prior or prolonged exposure to echinocandins, with a median time to onset of 100 days [4]. Nevertheless, rare cases of resistance can appear very quickly after echinocandins exposure [5], [6]. Here, we report a new case of rapid (12 days) acquisition of resistance to echinocandins in *C. glabrata*, associated to clinical failure in a colonized then infected patient. Comparison of isolates by multilocus sequence typing (MLST), results of *FKS* mutations and antifungal susceptibility testing of all isolates are reported. The interpretation of MIC values of *FKS* mutant strains is discussed and a screening strategy proposed.

## 2 Case report

Mr P., a 71-year-old man, was hospitalized for a second surgical treatment of a hiatal hernia (day 0). On day+2, the patient presented an acute respiratory distress syndrome accompanied by fever. He was intubated, ventilated and an empiric broad-spectrum antibiotic therapy was prescribed. His health condition worsened on day+16 with a resumption of sepsis. Blood cultures were positive with *Staphylococcus epidermidis* and *Streptococcus mitis*. The antibiotic therapy was reinforced with Piperacillin-Tazobactam, Vancomycin and Gentamicin. On day+22, the transoesophageal echocardiography did not show any cardiac vegetation and the CT scan did not detect any intra-abdominal infection. Nevertheless, as numerous risk factors for candidemia have been identified (supra-mesocolic surgery, broad-spectrum antibiotic therapy, hospital length of stay greater than 7 days, catheter), *Candida* colonization index was done. On day+23, mycological cultures of all samples (urine, anal, throat and gastric fluid samples) were positive with *C. glabrata*. A possible fungal infection after supra-mesocolic surgery was suspected and, in the presence of four colonized sites, micafungin (100 mg daily) was prescribed on day+25. A 2 mg/kg of methylprednisolone was also added to treat fibrosis phase of acute respiratory distress syndrome. After 12 days of administration, micafungin was switched for liposomal amphotericin B because of unfavourable clinical course with appearance of subcutaneous emphysema predominating in the right hemithorax and a right pneumothorax. Blood cultures remained negative. The patient's condition worsened and death occurred on day+45 in a context of sepsis and myocardial incompetence.

Peritoneal samples were taken immediately post-mortem, but no autopsy was performed. Mycological cultures showed numerous colonies of *C. glabrata* with two different color phenotypes (white and pink) on chromogenic medium (CHROMagar, Becton Dickinson). The four strains isolated at day+23, as well as the two colonies with a different phenotype isolated from the peritoneal fluid at day+45 were identified as *C. glabrata* by MALDI-TOF (VITEK® MS, Biomérieux and Multiflex, Bruker which allows distinction of C. *nivariensis* and C. *bracarensis*), and compared by MLST (Dodgson 2003). Sequences were analyzed on the website http://pubmlst.org/cglabrata/(accessed 11.01.2016). The sequence types (ST) of the isolates were identical (ST 7), which confirmed the persistence of the same strain in this patient. Antifungal susceptibility testing for amphotericin B, fluconazole, voriconazole, caspofungin, anidulafungin and micafungin was performed by Etest® method (Biomérieux), according to the manufacturer's instructions. MIC values were interpreted according to current Clinical and Laboratory Standards Institute (CLSI) breakpoints and epidemiological cutoff values (ECVs) [7]. *C. glabrata* isolates of day+45 presenting two different color phenotypes showed higher MIC values for echinocandin molecules than isolates of day+23. Sequencing of hot-spot 1 and 2 regions of the *FKS1* and *FKS2* genes [8], [9] brought out modifications of the *FKS2* gene for both isolates of day+45: a mutation (F659S) for one and a deletion (F659del) for the other one. No mutations of the *FKS* hotspots were found in the 4 *C. glabrata* strains isolated at day+23. Mycological results are presented in Table 1.

**Table 1:** Results of multi-locus sequence typing (MLST), MIC values and *FKS* mutations for all the isolates.

Strain	Origin	*FKS* genotype	MLST profile (ST^a^)	Etest MIC values (µg/mL)					
				CS	MICA	AND	FL	VO	AB
CG 743 (Day+23)^b^	Urine	WT	ST7	0,064	0,012	0,008	12	0.19	1
				(S)	(S)	(S)	(SDD)	(WT)	(WT)
CG 744	Anal swab	WT	ST7	0,094	0,012	0,008	12	0.25	0.75
(Day+23)				(S)	(S)	(S)	(SDD)	(WT)	(WT)
CG 745	Throat swab	WT	ST7	0,064	0,012	0,012	16	0.25	0.75
(Day+23)				(S)	(S)	(S)	(SDD)	(WT)	(WT)
CG 746	Gastric fluid	WT	ST7	0,064	0,008	0,008	12	0.19	0.75
(Day+23)				(S)	(S)	(S)	(SDD)	(WT)	(WT)
CG 849W	Peritoneal fluid	FKS2-F659S	ST7	0,38	0,032	0,047	12	0.25	0.75
(Day+45)				(I)	(S)	(S)	(SDD)	(WT)	(WT)
CG 864P	Peritoneal fluid	FKS2-F659del	ST7	>32	1,5	1	12	0.19	0.5
(Day+45)				(R)	(R)	(R)	(SDD)	(WT)	(WT)

CS: caspofungin, MICA: micafungin, AND: anidulafungin, FL: fluconazole, VO: voriconazole, AB: amphotericin B, S: sensible, I: intermediate, SDD: sensible dose-dependent, R: resistant, WT: wild-type

a ST=sequence type;

b Day of sampling

## 3 Discussion

The prevalence of strains with an echinocandin resistance remains low, around 4% for *C. glabrata* and less than 1% for *Candida albicans* [10]. However, the number of cases is increasing, particularly in *C. glabrata* where *FKS1* and *FKS2* mutations are responsible for reduced susceptibility to echinocandins and associated with therapeutic failure. Resistances usually appear after prior or prolonged exposure to echinocandins with a median time to onset of 100 days [4], but can appear very quickly after the prescription of echinocandins [5], [6]. Indeed, without prior exposure to echinocandins, a rapid emergence of *FKS* mutations, associated with therapeutic failure, was observed after 7 or 8 days of echinocandin treatment for *Candida kefyr* and *C. glabrata* respectively [5], [6], and after 12 days of micafungin in our case.

We found modifications of the *FKS2* gene in two isolates recovered from peritoneal fluid, a mutation (F659S) in one and a deletion (F659del) in the other. Changes in the *FKS2* gene encompass the majority of mutations, mainly in the Ser-663 and Phe-659 positions [11]. The deletion of phenylalanine at position 659 has already been associated with clinical failures [5]. The F659S mutation was also described, but the MICs of the strains containing this mutation were heterogeneous and no therapeutic failure has been reported [10], [11], [12].

Detection of resistance to echinocandins is carried out in daily practice by the determination of MIC values. The two reference methods are broth microdilution methods of the CLSI and the European Committee on Antimicrobial Susceptibility Testing (EUCAST). Since they are not usable on a daily practice, commercial techniques such as Etest® (Biomérieux) or Sensititre® (Trek Diagnostics) are used. However, interpretations of MIC values should be done with caution because clinical breakpoints lack harmonization, are not available for all *Candida* species and are not necessarily transposable to commercial techniques. Technique-specific ECVs could become the best way to interpret MIC values. Caspofungin susceptibility testing, although not recommended by CLSI and EUCAST, would allow a better screening of *FKS* mutant strains. This has been reported in two studies, where 89–100% of the mutant strains were intermediate or resistant to caspofungin *versus* 57–71% for micafungin and 71–78% for anidulafungin [10], [11]. In our case, with the application of CLSI breakpoints for MIC interpretation, all the echinocandins MIC values were interpreted as resistant for isolate with F659del, while only caspofungin MIC value was intermediate for isolate with F659S mutation. Regardless of the anti-fungal agent tested, an increase in MIC was observed for the mutant strains: from 4 to 6 times for the strain with F659S mutation and from 187 to 500 times for the strain with F659del. This corresponds to the observations of Prigent [12] for whom the F659S mutation appeared after 14 days of caspofungin treatment and gave echinocandins MICs 4 times higher than those of the wild-type strain. Thus, the F659S mutation is associated with a low-level resistance while the deletion in F659 has a high-level of resistance. However, as this two isolates were present in culture, they could both be involved in therapeutic failure.

Detection of echinocandin resistance in treated patients is necessary. For this purpose, it seems essential to repeat sampling sterile sites in order to recover the strains potentially resistant to antifungals. For candidemia, one blood culture per day until negativity is required to determine the end of candidemia [1], [2]. For other invasive candidiasis, additional controls should also be done at least every 2–3 days. The use of chromogenic medium allows the detection of *Candida* species, but also, as in our case report, the presence of different phenotypes for a same species with the same ST by MLST (see above). A determination of the MIC values for echinocandin molecules for isolates (and for each phenotype) from sterile sites is necessary. Furthermore, a repetition of antifungal susceptibility testing is essential when isolates are still recovered after one week of treatment. An increase in the MICs of at least 4 times, whatever the categorization of the strain (sensible, intermediate or resistant), must lead to the search for *FKS1* and *FKS2* mutations and to close monitoring of the patient. This strategy has the advantage of being free from the method used, the molecule tested and the knowledge of breakpoints or ECVs.

## Conflict of interest

There are none.
